# Biomarker-Stratified Efficacy of Immune Checkpoint Inhibitors in Locally Advanced Head and Neck Squamous Cell Carcinoma: A Systematic Review and Meta-Analysis of Randomized Trials

**DOI:** 10.3390/cancers18111679

**Published:** 2026-05-22

**Authors:** Ramaditya Srinivasmurthy, Daniel T. Jones, Rishi K. Nanda, Jason Ta, Abbas Hussain, Riccesha Hattin, Sisi Tian, Suparna Shah, Jo-Lawrence Bigcas, Robert Wang, Samual Francis, Kyaw Z. Thein

**Affiliations:** 1 Department of Internal Medicine, Mount Sinai Morningside/West, New York, NY 10019, USA; 2 Department of Internal Medicine, Sunrise Health GME Consortium, Las Vegas, NV 89128, USA; drdanielthomasjones@gmail.com; 3 College of Osteopathic Medicine, Touro University Nevada, Las Vegas, NV 89014, USA; rnanda24@gmail.com; 4 Department of Internal Medicine, HCA Healthcare/USF Morsani College of Medicine GME: HCA Florida Citrus Hospital, Inverness, FL 34452, USA; jasonta2016@gmail.com; 5 Department of Internal Medicine, Kirk Kerkorian School of Medicine at UNLV, Las Vegas, NV 89102, USA; abbasalihussain7@gmail.com (A.H.);; 6 Department of Otolaryngology, Kirk Kerkorian School of Medicine at UNLV, Las Vegas, NV 89102, USA; 7 Department of Otolaryngology Head and Neck Surgery, University of California San Diego School of Medicine, La Jolla, CA 92093, USA; 8 Division of Radiation Oncology, Comprehensive Cancer Centers of Nevada, Las Vegas, NV 89169, USA; 9 Division of Hematology and Medical Oncology, Comprehensive Cancer Centers of Nevada, Las Vegas, NV 89169, USA; kyaw.thein@usoncology.com

**Keywords:** LAHNSCC, ICI, immune checkpoint inhibitors, head and neck carcinoma, PD-L1, immunotherapy, locally advanced

## Abstract

**Introduction:** The role of immune checkpoint inhibitors (ICIs) in locally advanced head and neck squamous cell carcinoma (LA HNSCC) remains uncertain, with randomized trials showing inconsistent results in heterogeneous populations. We conducted a systematic review of randomized trials evaluating ICI-based strategies in LA HNSCC, with outcomes stratified by PD-L1 expression, HPV/p16 status, and cisplatin eligibility to identify patient subgroups most likely to benefit from ICIs. **Methods**: MEDLINE, Cochrane, and EMBASE databases were systematically searched up to 10 January 2026. Randomized controlled trials (RCTs) evaluating ICIs in patients with LA HNSCC were included. The primary outcome was pooled time-to-event efficacy, including event-free survival (EFS), progression-free survival (PFS), and disease-free survival (DFS) as reported across trials. A generic inverse variance method was used to calculate the estimated pooled hazard ratio (HR) for PFS with 95% confidence interval (CI). Heterogeneity was assessed with Cochran’s Q test. Random effects model was applied. **Results**: A total of 3605 patients from seven phase II/III RCTs were included. In the overall population, no significant difference in EFS/PFS/DFS was observed between ICI and standard therapy (HR 0.90; 95% CI: 0.77–1.06; *p* = 0.20). However, in subgroup analyses stratified by PD-L1 expression, patients with PD-L1-positive tumors demonstrated improved PFS with ICIs compared with control (HR 0.78; 95% CI: 0.67–0.91; *p* < 0.0001). In contrast, PD-L1-negative tumors demonstrated inferior PFS in the ICIs arm (HR 1.31; 95% CI: 1.02–1.68; *p* = 0.03). No significant differences in PFS were observed based on HPV or p16 status. A subset analysis of cisplatin-eligible LA HNSCC trials evaluating the addition of ICIs to standard therapy showed a similar pattern. ICI use in PD-L1-positive patients demonstrated significantly improved PFS (HR 0.76; 95% CI: 0.63–0.92; *p* < 0.0001), while ICI use in PD-L1-negative patients demonstrated decreased PFS (HR 1.28; 95% CI: 0.99–1.66; *p* = 0.06). In cisplatin-ineligible populations, ICI regimens did not improve PFS compared with cetuximab plus RT. **Conclusions**: This study showed that although in the overall population there was no significant difference in EFS/PFS/DFS, in the PD-L1-positive subgroup, patients experienced significantly improved PFS with ICIs compared with control, while in the PD-L1-negative subgroup, patients demonstrated inferior PFS in the ICIs arm; these results were mirrored in the cisplatin-eligible subgroup.

## Simple Summary

Although immune checkpoint inhibitors have shown clear benefit in patients with recurrent or metastatic head and neck cancer, their role in patients with locally advanced disease remains uncertain. This inconsistency may be due to differences in trial design and patient populations. To address this, we performed a systematic review and meta-analysis of randomized trials, examining outcomes based on programmed death-ligand 1 expression, human papillomavirus status, and eligibility for cisplatin-based chemotherapy. Overall, adding immune checkpoint inhibitors did not improve outcomes in the full study population. However, patients whose tumors expressed programmed death-ligand 1 experienced improved outcomes, in contrast to those without expression who had worse outcomes when these therapies were added. This pattern was also observed among patients eligible for cisplatin. These findings suggest that programmed death-ligand 1 is an important biomarker that can help guide treatment decisions and identify patients most likely to benefit from immunotherapy.

## 1. Introduction

Head and neck squamous cell carcinoma (HNSCC) accounts for approximately 6% of all cancer cases worldwide and contributes to nearly 3% of cancer-related mortality [1,2]. Approximately 60% of patients present with locally advanced disease (LA HNSCC) at diagnosis [2]. Curative-intent management involves multimodal therapy including surgery, radiation, and systemic treatment, most commonly cisplatin-based chemoradiation or cetuximab-based regimens in cisplatin-ineligible patients [3,4,5]. Outcomes remain limited, with relapse rates approaching 40% and 5-year overall survival near 50% [1]. Notably, patients with HPV-associated oropharyngeal disease demonstrate more favorable prognoses [1,2,6].

Immune checkpoint inhibitors (ICI) targeting the PD-1/PD-L1 axis have reshaped the treatment of recurrent and metastatic HNSCC (R/M HNSCC). These agents restore antitumor immune activity by blocking inhibitory signaling pathways that suppress T cell activation [7]. Randomized trials established survival benefit in the metastatic setting, supporting the incorporation of ICI into standard treatment paradigms and prompting investigation in curative-intent disease.

Randomized trials evaluating ICI in LA HNSCC have not demonstrated a consistent benefit. The phase III KEYNOTE-412 trial evaluating pembrolizumab with chemoradiation did not meet its primary endpoint of event-free survival (EFS) (hazard ratio 0.83; 95% confidence interval 0.68–1.03) [7]. The JAVELIN Head and Neck 100 trial evaluating avelumab with chemoradiotherapy was terminated early for futility with no improvement in progression-free survival (PFS) [8]. Additional studies evaluating maintenance strategies and radiotherapy-based regimens in cisplatin-ineligible populations have similarly failed to demonstrate improved outcomes [8,9,10,11].

Subgroup analyses suggest that the treatment effect may depend on tumor biology and patient selection. PD-L1 expression has been associated with improved outcomes in selected populations, with numerically greater benefit observed in PD-L1-positive subgroups in trials such as KEYNOTE-412 [7]. Patients with PD-L1-negative tumors demonstrate limited benefit and may experience inferior outcomes with ICI-based strategies [12]. Additional stratifiers, including HPV/p16 status and cisplatin eligibility, have biological relevance but demonstrate inconsistent predictive value across studies. Interpretation remains limited by heterogeneity in trial design, patient populations, and PD-L1 assessment methods, including combined positive score, tumor proportion score, and tumor area positivity.

A systematic evaluation of randomized evidence with biomarker-stratified outcomes is needed to clarify the role of ICIs in LA HNSCC. We conducted a systematic review and meta-analysis of randomized trials evaluating ICI-based strategies in the curative-intent setting, with outcomes stratified by PD-L1 expression, HPV/p16 status, and cisplatin eligibility to identify patient subgroups most likely to benefit from immunotherapy.

## 2. Methods

### 2.1. Search Strategy and Study Selection

The systematic review was conducted based on the Cochrane Handbook for Systematic Reviews and reported in accordance with the Preferred Reporting Items for Systematic Reviews and Meta-Analyses (PRISMA) guidelines [13,14]. A comprehensive literature search was performed using MEDLINE, Cochrane, and EMBASE from database inception through 10 January 2026. The search strategy incorporated combinations of the following terms: “head and neck squamous cell carcinoma,” “locally advanced,” “immunotherapy,” “immune checkpoint inhibitor,” “PD-1,” and “PD-L1.” Full Boolean strings can be found in the Supplementary Materials, Table S1A–C.

Eligible studies included phase II and phase III randomized controlled trials evaluating immune checkpoint inhibitor (ICI)-based strategies in the curative-intent setting of locally advanced HNSCC. Trials conducted in the recurrent or metastatic setting, non-randomized studies, and studies lacking survival outcomes were excluded.

**Figure 1:**
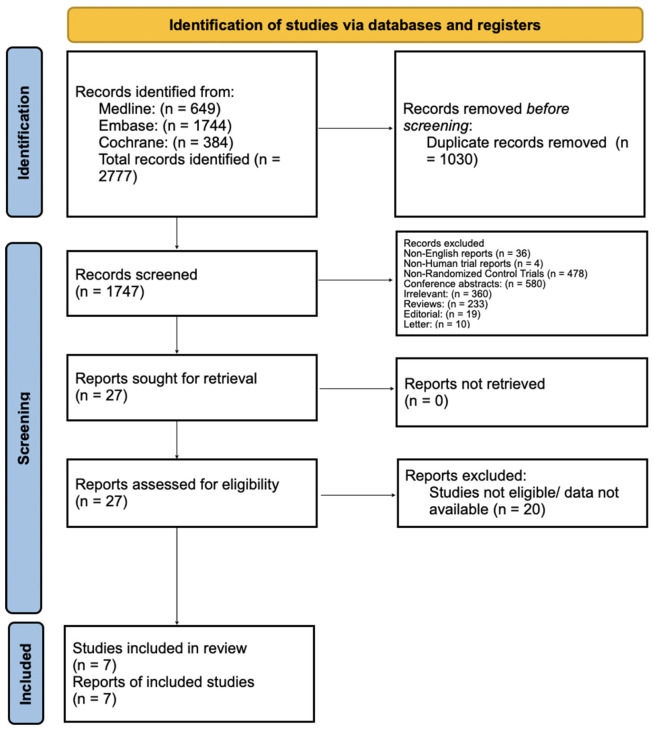
PRISMA Flow Diagram: Flow diagram summarizing database search results, duplicate removal, screening, eligibility assessment, and final inclusion of randomized trials in the systematic review and meta-analysis.

Two reviewers independently screened titles and abstracts, followed by full-text review. Discrepancies and biases were resolved by consensus with the involvement of other reviewers. Study selection was documented using a PRISMA flow diagram (Figure 1).

### 2.2. Data Extraction and Outcomes

Data extraction was performed independently by two reviewers using a standardized data collection framework. Extracted variables included trial characteristics (phase, population, treatment arms, sample size), treatment setting (definitive, perioperative, or maintenance), and reported outcomes.

The primary outcome was event-free survival (EFS) or analogous time-to-event endpoints, such as progression-free survival (PFS) and disease-free survival (DFS), depending on trial reporting. EFS generally captures progression, recurrence, treatment failure, or death, while PFS and DFS represent related time-to-event measures used across included trials. EFS was defined as the time from randomization to a protocol-defined event, which could include disease progression, recurrence, treatment failure, inability to proceed with planned definitive therapy, or death, depending on the trial context. PFS was defined as the time from randomization or treatment initiation to radiographic or clinical disease progression or death. DFS was defined as the time after completion of curative-intent therapy, most commonly surgery or definitive treatment, to disease recurrence or death. Because included trials evaluated different treatment settings, including definitive chemoradiation, perioperative therapy, and maintenance therapy, the specific events included within EFS, PFS, and DFS varied across studies. These endpoints were therefore pooled as related but non-identical time-to-event efficacy outcomes reflecting failure to maintain disease control, and pooled estimates were interpreted in the context of this clinical and methodological heterogeneity. Overall survival (OS) was evaluated as a secondary outcome when available. Hazard ratios (HRs) with corresponding 95% confidence intervals (CIs) were extracted preferentially.

Risk of bias was independently assessed by two reviewers using the Cochrane Risk of Bias 2 (RoB 2) tool for randomized trials. Domains evaluated included randomization process, deviations from intended interventions, missing outcome data, measurement of outcomes, and selection of reported results. Discrepancies were resolved by consensus. Publication bias was visually assessed using funnel plots, which are provided in Figure S1. Study-level risk of bias was assessed using the Cochrane Risk of Bias 2 tool, with the risk of bias graph and summary provided in Figure S2 and Figure S3, respectively.

### 2.3. Trial and Biomarker Stratification

Included studies were categorized based on treatment setting to account for biological and clinical heterogeneity: (1) definitive chemoradiation-based strategies in unresectable disease, (2) perioperative strategies including neoadjuvant and/or adjuvant immunotherapy in resectable disease, and (3) maintenance or post-definitive approaches following completion of multimodal therapy.

Biomarker-stratified analyses were conducted according to PD-L1 expression, HPV/p16 status, and cisplatin eligibility. PD-L1 positivity was defined according to trial-specific criteria, including combined positive score (CPS), tumor proportion score (TPS), or tumor area positivity (TAP), with corresponding thresholds as reported in each study. HPV or p16 status was extracted based on immunohistochemistry or trial-defined classification. Cisplatin eligibility was determined according to trial inclusion criteria and stratified accordingly. A structured comparison of PD-L1 assay methodologies and thresholds across included trials is provided in Supplementary Table S2.

Given variability in biomarker definitions and assay methodologies across trials, subgroup analyses were interpreted within the context of reported thresholds and study-specific definitions.

Although included trials differed in treatment setting, all evaluated ICI-based strategies in the curative-intent LA HNSCC setting and reported time-to-event efficacy outcomes. Trials were, therefore, pooled to evaluate the overall association between ICI addition and disease-control outcomes across randomized curative-intent studies, with subgroup and limitation analyses used to contextualize clinical and methodological heterogeneity.

### 2.4. Statistical Analysis

Meta-analysis was performed using a random-effects model to account for clinical and methodological heterogeneity across studies. Hazard ratios and corresponding standard errors were pooled using the generic inverse variance method. EFS, PFS, and DFS were considered similar time-to-event efficacy endpoints and were pooled for analysis, given differences in endpoint reporting across included trials.

Heterogeneity was assessed using Cochran’s Q test and quantified with the I^2^ statistic, with values greater than 50% considered indicative of substantial heterogeneity. All analyses were conducted using Review Manager (RevMan, version 5.3). Statistical significance was defined as a two-sided *p*-value less than 0.05.

The primary outcomes of interest, including PFS, EFS, and DFS, were specified prior to data extraction. Subgroup analyses stratified by PD-L1 expression, HPV/p16 status, and cisplatin eligibility were performed post hoc based on the availability of reported subgroup data across included trials. When data were missing or not reported, no imputation was performed, and analyses were restricted to available reported data. A formal assessment of the certainty of evidence was not performed due to heterogeneity in study design and endpoints.

This review was not prospectively registered in PROSPERO, and a formal protocol was not prepared prior to study initiation. This reflects the exploratory nature of the analysis and the intent to synthesize emerging randomized evidence in a rapidly evolving field.

## 3. Results

A total of 3605 patients from seven randomized controlled trials were included, comprising five phase III trials and two phase II trials. Key characteristics of the included studies are summarized in Table S3 in the Supplementary Materials. All studies evaluated ICI-based strategies versus standard therapy in LA HNSCC, with heterogeneity in treatment setting and timing of ICI administration.

**Figure 2:**
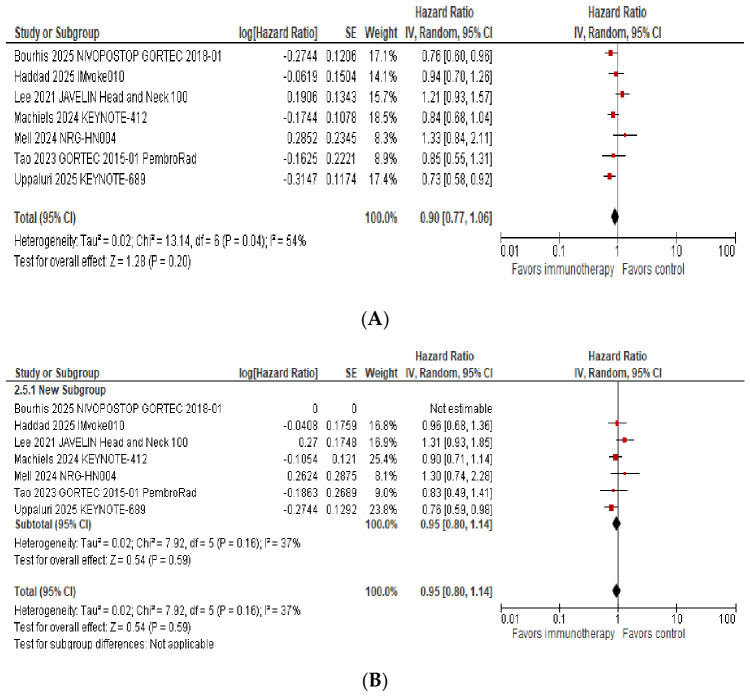
(**A**) Forest plot of pooled time-to-event efficacy outcomes in the overall study population. Pooled progression-free survival, event-free survival, and disease-free survival outcomes are shown for immune checkpoint inhibitor-based strategies versus standard therapy [7,8,9,10,11,16,17]. (**B**) Forest plot of overall survival in the overall study population. Pooled overall survival outcomes are shown for immune checkpoint inhibitor-based strategies versus standard therapy [7,8,9,10,11,16,17].

**Figure 3:**
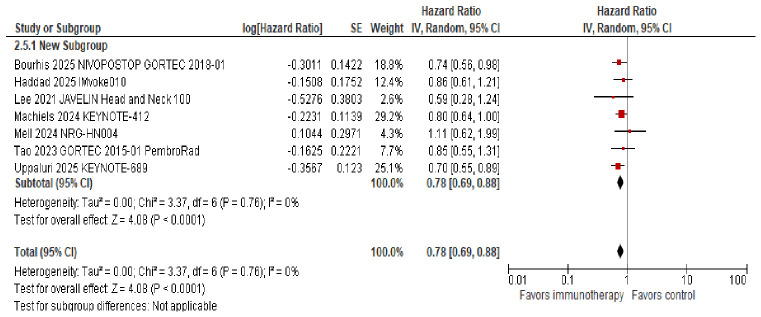
Forest plot of pooled time-to-event outcomes in patients with PD-L1-positive tumors. Pooled progression-free survival, event-free survival, and disease-free survival outcomes are shown for immune checkpoint inhibitor-based strategies versus standard therapy [7,8,9,10,11,16,17].

**Figure 4:**
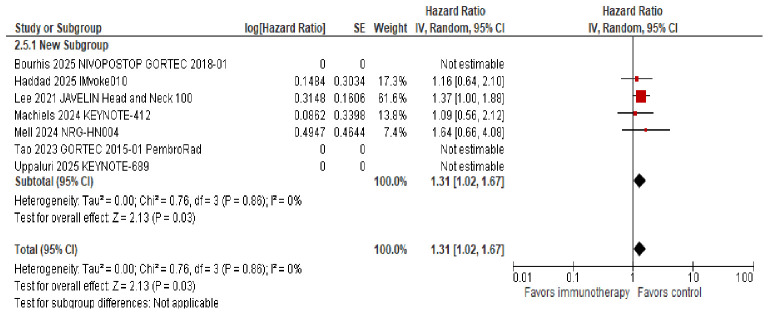
Forest plot of pooled time-to-event outcomes in patients with PD-L1-negative tumors. Pooled progression-free survival, event-free survival, and disease-free survival outcomes are shown for immune checkpoint inhibitor-based strategies versus standard therapy in PD-L1-negative subgroups [7,8,9,10,11,16,17].

**Figure 5:**
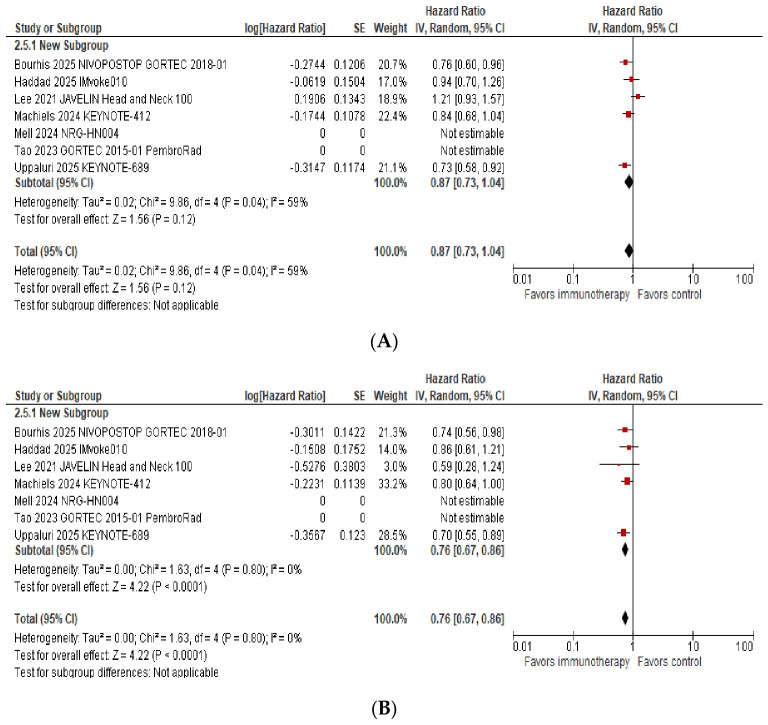

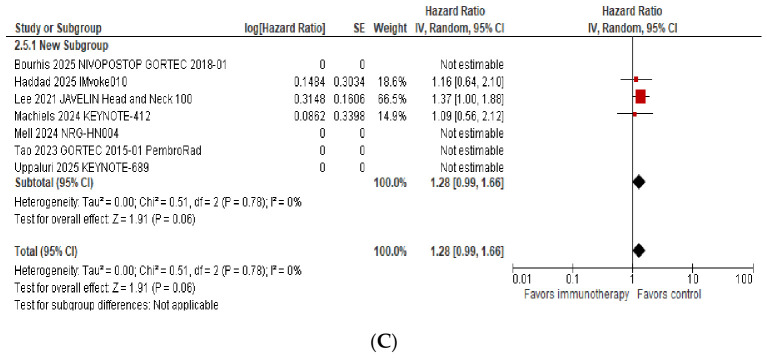
(**A**) Forest plot of pooled time-to-event outcomes in the cisplatin-eligible population. Pooled progression-free survival, event-free survival, and disease-free survival outcomes are shown for immune checkpoint inhibitor-based strategies versus standard therapy among cisplatin-eligible trial populations [7,8,9,10,11,16,17]. (**B**) Forest plot of pooled time-to-event outcomes in PD-L1-positive cisplatin-eligible patients. Pooled progression-free survival, event-free survival, and disease-free survival outcomes are shown for immune checkpoint inhibitor-based strategies versus standard therapy [7,8,9,10,11,16,17]. (**C**) Forest plot of pooled time-to-event outcomes in PD-L1-negative cisplatin-eligible patients. Pooled progression-free survival, event-free survival, and disease-free survival outcomes are shown for immune checkpoint inhibitor-based strategies versus standard therapy [7,8,9,10,11,16,17].

**Figure 6:**
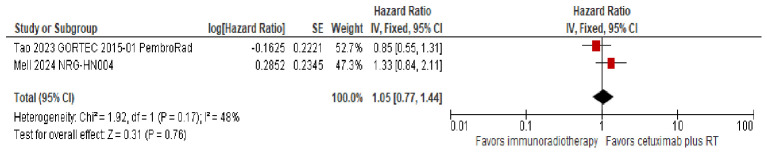
Forest plot of pooled time-to-event outcomes in cisplatin-ineligible patients. Pooled progression-free survival, event-free survival, and disease-free survival outcomes are shown for immune checkpoint inhibitor plus radiotherapy versus cetuximab plus radiotherapy [10,11].

**Figure 7:**
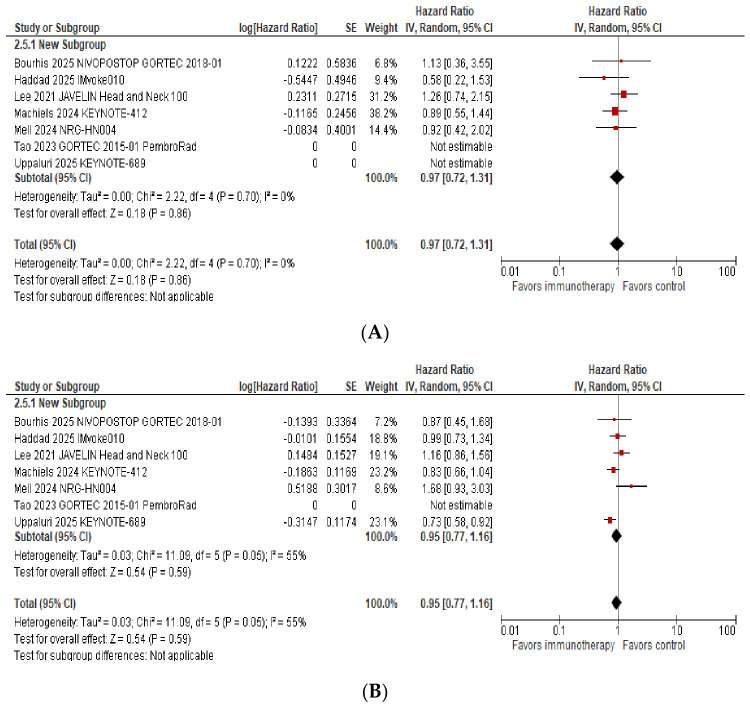
(**A**) Forest plot of pooled time-to-event outcomes in HPV/p16-positive patients. Pooled progression-free survival, event-free survival, and disease-free survival outcomes are shown for immune checkpoint inhibitor-based strategies versus standard therapy [7,8,9,10,11,16,17]. (**B**) Forest plot of pooled time-to-event outcomes in HPV/p16-negative patients. Pooled progression-free survival, event-free survival, and disease-free survival outcomes are shown for immune checkpoint inhibitor-based strategies versus standard therapy [7,8,9,10,11,16,17].

Two trials evaluated ICIs combined with definitive radiotherapy in cisplatin-ineligible patients (NRG-HN004 [10] and GORTEC 2015 PembroRad [11]). Two phase III trials assessed the addition of ICIs to cisplatin-based chemoradiotherapy in cisplatin-eligible populations (KEYNOTE-412 [7] and JAVELIN Head and Neck 100 [8]). Two additional trials evaluated perioperative or postoperative ICI strategies in resectable disease (KEYNOTE-689 [15] and NIVOPOSTOP GORTEC 2018 [16]), and IMvoke010 [9] investigated atezolizumab maintenance following definitive therapy. Across studies, endpoints were reported as PFS, EFS, or DFS and were pooled for analysis. Subgroup data based on PD-L1 expression, HPV/p16 status, and cisplatin eligibility were extracted when available. Figure 2, Figure 3, Figure 4, Figure 5, Figure 6 and Figure 7 provide forest plots summarizing our findings.

In the pooled analysis of the overall study population, there was no statistically significant difference in pooled time-to-event outcomes (EFS/PFS/DFS) between patients treated with ICI-based strategies and those receiving standard therapy (hazard ratio [HR] 0.90; 95% confidence interval [CI]: 0.77–1.06; *p* = 0.20, I^2^: 54%) (Figure 2A). Overall survival data were immature at the time of analysis and did not demonstrate a significant difference between treatment arms (HR: 0.95; 95% CI: 0.80–1.14; *p* = 0.59, I^2^: 37%) (Figure 2B). Median follow-up varied across included trials, ranging from approximately 16.7 months in JAVELIN Head and Neck 100 to 47.7 months in KEYNOTE-412, as summarized in Supplementary Table S3.

In subgroup analyses stratified by PD-L1 expression, a differential treatment effect was observed. Among patients with PD-L1-positive tumors, defined according to trial-specific thresholds (PD-L1 CPS ≥1, PD-L1 tumor area positivity ≥5%, or PD-L1 tumor proportion score ≥25%), treatment with ICI was associated with a significant improvement in pooled time-to-event outcomes compared with standard therapy (HR 0.78; 95% CI: 0.69–0.88; *p* < 0.0001; I^2^ = 0%) (Figure 3).

In contrast, among patients with PD-L1-negative tumors, ICI-based treatment was associated with inferior EFS/PFS/DFS compared with control therapy (HR 1.31; 95% CI: 1.02–1.67; *p* = 0.03, I^2^: 0%) (Figure 4). Data for PD-L1-negative subgroups were not available for all trials, including GORTEC 2015-01 PembroRad, KEYNOTE-689, and NIVOPOSTOP GORTEC 2018-01 [11,15,16]. Across included trials with available data, a total of n = 2590 patients contributed to the PD-L1-positive pooled estimate and n = 795 patients to the PD-L1-negative pooled estimate (graphical representation can be found in Supplementary Materials Table S2). Notably, PD-L1-negative subgroups comprised a substantially smaller proportion of the overall study populations across trials, often representing less than 10% of enrolled patients.

A subset analysis restricted to cisplatin-eligible LA HNSCC trials, including KEYNOTE-412, JAVELIN Head and Neck 100, KEYNOTE-689, NIVOPOSTOP GORTEC 2018-01, and IMvoke010 [7,9,11], demonstrated a pattern consistent with the overall PD-L1-stratified findings. In the overall cisplatin-eligible population, ICI-based strategies did not result in a statistically significant improvement in pooled time-to-event outcomes (EFS/PFS/DFS) compared with standard therapy (HR 0.87; 95% CI: 0.73–1.04; *p* = 0.12, I^2^: 59%) (Figure 5A).

Among PD-L1-positive cisplatin-eligible patients, treatment with ICIs was associated with a significant improvement in pooled time-to-event outcomes (HR 0.76; 95% CI: 0.67–0.86; *p* < 0.0001, I^2^: 0%) (Figure 5B). Conversely, in PD-L1-negative cisplatin-eligible patients, ICI use was associated with a numerically worse pooled time-to-event outcomes, which did not reach statistical significance (HR 1.28; 95% CI: 0.99–1.66; *p* = 0.06, I^2^: 0%) (Figure 5C).

In cisplatin-ineligible populations, limited to NRG-HN004 and GORTEC 2015-01 PembroRad, the use of ICIs combined with radiotherapy did not improve EFS/PFS/DFS compared with cetuximab plus radiotherapy (HR 1.05; 95% CI: 0.77–1.44; *p* = 0.78, I^2^: 48%) (Figure 6). Subgroup analyses based on PD-L1 expression were not feasible due to limited data availability.

Subgroup analyses stratified by HPV or p16 status did not demonstrate a statistically significant difference in pooled time-to-event outcomes between ICI-based therapy and standard treatment. Among patients with HPV/p16-positive disease, pooled analysis showed no significant benefit with ICIs (HR 0.97; 95% CI: 0.72–1.31; *p* = 0.86, I^2^: 0%) (Figure 7A). Similarly, in HPV/p16-negative populations, ICI-based therapy was not associated with improved outcomes compared with control treatment (HR 0.95; 95% CI: 0.77–1.16; *p* = 0.59, I^2^: 55%) (Figure 7B). Interpretation of HPV/p16-specific effects remains limited by incomplete subgroup reporting across trials and imbalances in HPV prevalence among included study populations.

## 4. Discussion

### 4.1. Principal Findings

In this analysis of randomized trials evaluating ICI-based strategies in LA HNSCC, the addition of ICIs to standard curative-intent therapy did not result in a statistically significant improvement in survival outcomes in the overall patient population. Across the overall pooled cohort, ICI-based approaches failed to improve time-to-event outcomes (EFS/PFS/DFS) compared with standard therapy, and overall survival data remained immature without evidence of a meaningful difference between treatment arms.

However, when prespecified subgroups were analyzed, the data revealed a differential treatment effect according to tumor PD-L1 expression. Among patients with PD-L1-positive tumors, ICI-based strategies were associated with a significant improvement in outcomes compared with standard therapy. Inversely, patients with PD-L1-negative tumors experienced inferior outcomes with the addition of ICIs, with a signal towards worse PFS, EFS, and DFS relative to control therapy. Although subgroup data were not uniformly available across all trials, the consistency of this pattern across multiple studies suggests that PD-L1 expression may modify treatment effect in the curative-intent setting.

Importantly, this PD-L1-stratified pattern was preserved in analyses restricted to cisplatin-eligible populations, reinforcing the observed effect modification and reducing the likelihood that the findings are driven solely by differences in baseline treatment intensity. No convincing benefit of ICI-based strategies was observed in cisplatin-ineligible populations treated with radiotherapy-based regimens. In contrast to PD-L1, HPV/p16 status was not associated with differential treatment effect in the available subgroup analyses. However, this finding should be interpreted cautiously, as HPV/p16-stratified analyses were limited by small sample sizes, incomplete reporting across trials, and imbalances in HPV prevalence among included study populations. Therefore, the present analysis does not establish HPV/p16 status as a predictive biomarker for ICI benefit or risk in LA HNSCC, but incomplete subgroup reporting prevents definitive conclusions regarding its predictive role.

### 4.2. Biological and Clinical Interpretation

Historically, platinum-based chemotherapy with cetuximab represented a standard first-line systemic therapy approach in recurrent/metastatic HNSCC [17,18]. More recently, PD-L1 expression has become the most clinically established biomarker for identifying patients with recurrent/metastatic HNSCC who are more likely to benefit from PD-1 blockade with agents such as pembrolizumab and nivolumab. Landmark trials, including KEYNOTE-048 and CheckMate-141 [19,20], demonstrated a survival benefit with PD-1-based strategies in the R/M HNSCC setting. In contrast, trials evaluating ICI-based strategies in LA HNSCC, including KEYNOTE-412, JAVELIN Head and Neck 100, and IMvoke010 [7,8,9] have not consistently demonstrated the same degree of benefit. This contrast with KEYNOTE-048 is clinically important, as pembrolizumab-based therapy improved overall survival in R/M HNSCC, particularly among patients with PD-L1-expressing tumors, supporting PD-L1 as a clinically relevant biomarker in that setting [21,22].

Several biological and therapeutic differences may explain why ICI benefit has not translated uniformly from R/M HNSCC to the locally advanced setting. Recurrent/metastatic tumors often arise after prior exposure to surgery, radiation, and/or systemic therapy and may, therefore, exist within a more immune-modified tumor microenvironment (TME) characterized by adaptive immune resistance, PD-1/PD-L1 pathway activation, and exhausted tumor infiltrating lymphocytes. In this context, checkpoint blockade may restore pre-existing but functionally suppressed antitumor immunity. In contrast, locally advanced disease treated with curative intent may include tumors with lower systemic tumor burden, different antigenic exposure, and greater heterogeneity in baseline immune infiltration. As a result, PD-L1 expression may identify tumors with an already primed but inhibited immune microenvironment that is more susceptible to PD-1/PD-L1 blockade, whereas PD-L1-negative tumors may lack sufficient pre-existing antitumor immune activity to benefit from checkpoint inhibition [23,24].

Treatment sequencing may also influence ICI efficacy in locally advanced disease. In definitive chemoradiation strategies, ICIs are administered concurrently with radiation and cytotoxic chemotherapy, both of which can have opposing immunologic effects. Radiation may promote tumor antigen release, dendritic cell activation, T cell priming, and inflammatory signaling, potentially enhancing response to checkpoint blockade. However, radiation and chemotherapy may also induce lymphodepletion, impair circulating and intratumoral effector T cell populations, increase tissue inflammation, and promote immunosuppressive remodeling within the tumor microenvironment [23,24,25]. Similarly, surgery can reduce tumor antigen burden and alter the immune contexture before adjuvant immunotherapy, while maintenance strategies given after completion of definitive therapy may occur when residual antigenic stimulation is limited. These setting-specific differences may help explain why ICI efficacy varies across definitive, perioperative, postoperative, and maintenance approaches rather than producing a uniform benefit across all LA HNSCC populations.

Therefore, the efficacy of ICIs in LA HNSCC likely depends not only on biomarker status but also on the timing of immune checkpoint blockade relative to radiation, chemotherapy, surgery, and residual tumor antigen burden. The positive results of KEYNOTE-689 suggest that timing and patient selection may be critical, as perioperative pembrolizumab improved EFS in resectable LA HNSCC, supporting the need for biomarker-driven and specific treatment setting interpretation rather than uniform application of ICIs across all LA HNSCC populations [15].

This lack of consistent benefit observed with ICIs in unselected LA HNSCC differs from the established efficacy of PD-1 pathway blockade in the recurrent/metastatic setting and may reflect fundamental biological differences between these disease states. RM HNSCC tumors often utilize immune evasion pathways such as upregulation of PD-1/PD-L1 reflecting a TME where antitumor immune responses are already present but functionally disabled by inhibitory signaling [8]. In this context, checkpoint blockade may effectively reinvigorate exhausted T cell populations and restore antitumor activity. In contrast, the curative-intent setting of locally advanced disease is characterized by different tumor burdens, patterns of TME, and host immune dynamics, which may not be optimally aligned with the mechanisms through which PD-1 blockade exerts its effects.

### 4.3. Implications for Patient Selection and Trial Design

Our findings underscore the importance of PD-L1 stratification in guiding the use of ICI-based strategies. Patients with PD-L1-positive tumors consistently derived benefit from ICI addition, whereas those with PD-L1-negative disease experienced inferior outcomes, including worsened PFS, EFS, and DFS compared with standard therapy alone. This pattern persisted within the cisplatin-eligible population, reinforcing the robustness of this effect across treatment-eligible groups. In contrast, no meaningful differences were observed across HPV or p16-defined subgroups, suggesting that these factors should not influence decisions regarding ICI incorporation.

From a clinical standpoint, these findings support assessment of PD-L1 expression when ICI-based therapy is being considered for patients with LA HNSCC, particularly in perioperative or clinical trial settings [15,26,27,28]. Given its established clinical use in HNSCC, PD-L1 immunohistochemistry using combined positive score (CPS) may be the most practical approach when available, although treatment decisions should remain specific to the setting and should not rely on PD-L1 status alone [26,28,29]. In particular, PD-L1-negative status should not be interpreted as definitive evidence of harm, but should prompt extra caution when considering ICI intensification outside established indications or clinical trials. Prospective studies should continue to incorporate standardized PD-L1 testing and stratification to clarify which LA HNSCC populations derive durable benefit from immunotherapy.

### 4.4. Limitations and Strengths

This study has several important limitations. First, there was substantial clinical and methodological heterogeneity across the included trials. Studies differed in treatment setting (definitive chemoradiation, perioperative therapy, postoperative therapy, and maintenance therapy), patient population (resectable versus unresectable disease and cisplatin-eligible versus cisplatin-ineligible cohorts), control regimens, ICI agents, and timing of ICI administration. Primary tumor sites, including oropharynx, larynx, hypopharynx, and oral cavity, were also not consistently reported or stratified across studies, representing an additional source of unaccounted variability given known biological and prognostic differences between subsites. Although random-effects models were used to account for study heterogeneity, these differences limit the interpretability of the pooled estimates and support cautious interpretation of the overall results.

Second, the pooled efficacy analysis combined related but non-identical time-to-event endpoints of EFS, PFS, and DFS. These endpoints each reflect failure to maintain disease control but differ in event definitions and clinical context. EFS may include broader protocol-specific events such as disease progression, recurrence, treatment failure, inability to proceed with planned definitive therapy, or death. PFS primarily captures progression or death, whereas DFS is generally applied after curative-intent therapy and captures recurrence or death. These distinctions are particularly relevant because the included trials span definitive, perioperative, postoperative, and maintenance strategies. Therefore, the pooled estimate should be interpreted as a synthesis of related time-to-event efficacy outcomes rather than a uniform endpoint.

Third, PD-L1 assessment was heterogeneous across trials. KEYNOTE-412 and KEYNOTE-689 used a CPS threshold of ≥1, GORTEC 2015-01 PembroRad used tumor area positivity (TAP) ≥5%, and NIVOPOSTOP GORTEC 2018-01 used tumor proportion score (TPS) ≥25% [26]. These scoring systems measure related but non-identical aspects of PD-L1 expression. CPS incorporates PD-L1 expression on tumor and immune cells relative to the total number of viable tumor cells, TPS reflects PD-L1 expression on tumor cells alone, and TAP evaluates the proportion of tumor area demonstrating PD-L1 staining [26,27]. Therefore, classification of tumors as “PD-L1 positive” was based on trial-specific criteria rather than a uniform biomarker definition. As a result, PD-L1-positive subgroups were not assumed to be biologically or clinically equivalent across trials. A patient classified as PD-L1-positive by one scoring system or threshold may not necessarily meet positivity criteria under another system. This creates potential for subgroup misclassification, limits cross-trial comparability, and may influence the magnitude of pooled treatment effects. Therefore, PD-L1 subgroup analyses in this study were based on trial-defined categories and should be interpreted as exploratory. However, despite this variability, PD-L1-stratified analyses demonstrated a consistent directional treatment effect across trials, with improved outcomes observed in PD-L1-positive populations and no clear benefit, and in some analyses, worse outcomes, in PD-L1-negative subgroups. These findings suggest that PD-L1 expression retains biological relevance as a predictive biomarker in this setting, although the magnitude of benefit may vary depending on the assay and threshold used [30].

Fourth, the analysis was limited by the use of trial-level data rather than individual patient data. This prevented adjustment for patient-specific confounders, including tumor site, HPV/p16 status, smoking history, disease stage, performance status, treatment adherence, and other prognostic variables. Subgroup analyses were also limited by incomplete reporting across trials, particularly for PD-L1-negative populations. The PD-L1-negative subgroup included substantially fewer patients than the PD-L1-positive subgroup (n = 795 vs. n = 2590) and was unavailable in several key trials, limiting statistical power and increasing the instability of effect estimates. Missing PD-L1-negative subgroup data may also introduce selection bias, reduce the reliability of the pooled estimate, and limit the generalizability of findings to the broader PD-L1-negative LA HNSCC population. Therefore, the observed inferior outcomes in PD-L1-negative patients should be interpreted cautiously as a hypothesis-generating signal rather than definitive evidence of harm from ICI-based therapy.

Fifth, overall survival data remain immature for several included studies, limiting conclusions regarding long-term clinical benefit. Longer follow-up is needed to determine whether the observed subgroup differences in EFS, PFS, and DFS translate into durable overall survival advantages or harms.

Overall, despite these limitations, this study has several strengths. It synthesizes randomized evidence across the evolving curative-intent LA HNSCC immunotherapy landscape, incorporates biomarker-stratified analyses, evaluates clinically relevant subgroups including PD-L1 expression, HPV/p16 status, and cisplatin eligibility, and explicitly addresses heterogeneity using random-effects models and structured sensitivity-oriented interpretation. The consistency of treatment effect by PD-L1 status across available studies supports further prospective evaluation of PD-L1-guided treatment strategies in locally advanced HNSCC.

## 5. Future Directions

Future studies should prioritize prospective biomarker-stratified randomized trials to validate PD-L1-guided treatment strategies in the locally advanced setting. These trials should incorporate standardized PD-L1 assay protocols, synchronized scoring systems, and predefined cutoff definitions to improve trial comparability and clinical applicability [28,29]. Future individual patient data meta-analyses may help standardize PD-L1 scoring methodologies and threshold definitions across trials, allowing for more precise assessment of biomarker-defined treatment effects. Standardized biomarker reporting and patient-level correlative analyses will be important to better define the predictive role of PD-L1 in the locally advanced setting. Given the potential for harm in PD-L1-negative populations, biomarker trial designs or stratified randomization approaches are warranted. Additionally, exploration of other biomarkers, including tumor immune microenvironment signatures, may further refine patient selection. Longer follow-up is also needed to determine whether these subgroup effects translate into durable overall survival differences.

## 6. Conclusions

Our analysis demonstrates that when treatment effects are evaluated within biologically relevant subgroups, a consistent pattern of effect modification by PD-L1 expression emerges across trials in the locally advanced setting. PD-L1 expression appears to be associated with differential treatment effect across trials, with PD-L1-positive subgroups demonstrating improved pooled outcomes and PD-L1-negative subgroups demonstrating inferior outcomes with ICI-based strategies. These findings should be interpreted cautiously, as they are derived from trial-level subgroup analyses and do not establish causality, but may reflect underlying biological effect modification. In contrast, HPV/p16 status was not associated with differential treatment effect and does not appear to be a useful biomarker for guiding ICI use in this context. Taken together, our findings underscore the importance of biomarker-driven patient selection and warrant prospective validation of PD-L1-guided treatment strategies in the locally advanced setting.

## Data Availability

Analytic outputs (including RevMan files) are available from the corresponding author upon reasonable request.

## Supplementary Materials

The following supporting information can be downloaded at: https://www.mdpi.com/article/10.3390/cancers18111679/s1, Figure S1: Funnel Plot of Comparison. Funnel plot assessing potential publication bias among included studies for the pooled efficacy outcome; Figure S2: Risk Of Bias Graph. Risk of bias graph summarizing the overall percentage of studies judged as low, unclear, or high risk of bias across each Cochrane risk-of-bias domain; Figure S3: Risk of Bias Summary. Risk of bias summary showing domain-level judgments for each included randomized controlled trial; Table S1: The complete search strategies used for MEDLINE (1A), Embase (1B), and Cochrane (1C) are pro-vided below. Search terms were adapted for each database while preserving the same core con-cepts: disease population, immune checkpoint inhibitor exposure, locally advanced treatment setting, and randomized/phase II–III study design; Table S2: Summary of PD-L1 subgroup definitions and the number of PD-L1–positive and PD-L1–negative patients reported across included trials; Table S3: Key characteristics of included randomized controlled trials, including study phase, population, sample size, intervention, control arm, treatment setting, primary endpoint, and PD-L1 positivity definition;.

## Abbreviations

The following abbreviations are used in this manuscript:

ICI	Immune checkpoint inhibitors
LA HNSCC	Locally advanced head and neck squamous cell carcinoma
PD-L1	Programmed death-ligand 1
HPV	Human papillomavirus
RCTs	Randomized controlled trials
PFS	Progression-free survival
HR	Hazard ratio
EFS	Event-free survival
DFS	Disease-free survival
RT	Radiotherapy
HNSCC	Head and neck squamous cell carcinoma
R/M HNSCC	Recurrent and metastatic HNSCC
TME	Tumor microenvironment
CPS	Combined positive score
TAP	Tumor area positivity
TPS	Tumor proportion score
