# Supplementary material for: Biomarker-Stratified Efficacy of Immune Checkpoint Inhibitors in Locally Advanced Head and Neck Squamous Cell Carcinoma: A Systematic Review and Meta-Analysis of Randomized Trials
Source: Cancers (Basel). 2026 May 22;18(11):1679. doi: 10.3390/cancers18111679 (PMC13255863; doi:10.3390/cancers18111679)
Supplement: Supplementary file 1 [file cancers-18-01679-s001.zip › cancers-4289712-supplementary.pdf]

Supplementary Table S1:
The complete search strategies used for MEDLINE (1A), Embase (1B), and Cochrane (1C) are provided below. Search terms were adapted for each database while preserving the same core concepts: disease population, immune checkpoint inhibitor exposure, locally advanced treatment setting, and randomized/phase II–III study design.

MEDLINE 1A:

Search concept	Search terms
Population	"Head and Neck Neoplasms"[Mesh] OR "head and neck squamous cell carcinoma" OR HNSCC OR LA-HNSCC
Intervention	"Immune Checkpoint Inhibitors"[Mesh] OR immunotherapy OR "immune checkpoint inhibitor*" OR pembrolizumab OR nivolumab OR durvalumab OR avelumab OR atezolizumab OR anti-PD-1 OR anti-PD-L1
Disease setting	locally advanced OR resectable OR unresectable OR perioperative OR neoadjuvant OR adjuvant OR chemoradiotherapy
Study design	randomized controlled trial[pt] OR randomized OR randomised OR phase II OR phase III
Final search strategy	("Head and Neck Neoplasms"[Mesh] OR "head and neck squamous cell carcinoma" OR HNSCC OR LA-HNSCC) AND ("Immune Checkpoint Inhibitors"[Mesh] OR immunotherapy OR "immune checkpoint inhibitor*" OR pembrolizumab OR nivolumab OR durvalumab OR avelumab OR atezolizumab OR anti-PD-1 OR anti-PD-L1) AND (locally advanced OR resectable OR unresectable OR perioperative OR neoadjuvant OR adjuvant OR chemoradiotherapy) AND (randomized controlled trial[pt] OR randomized OR randomised OR phase II OR phase III)

Embase 1B:

Search concept	Search terms
Population	'head and neck squamous cell carcinoma' OR HNSCC OR 'head and neck cancer'
Intervention	pembrolizumab OR nivolumab OR durvalumab OR avelumab OR atezolizumab OR 'immune checkpoint inhibitor*' OR immunotherapy OR 'PD-1' OR 'PD-L1'
Disease setting	'locally advanced' OR resectable OR unresectable OR neoadjuvant OR adjuvant OR perioperative OR chemoradiotherapy
Study design	randomized OR randomised OR 'phase II' OR 'phase III'
Final search strategy	('head and neck squamous cell carcinoma' OR HNSCC OR 'head and neck cancer') AND (pembrolizumab OR nivolumab OR durvalumab OR avelumab OR atezolizumab OR 'immune checkpoint inhibitor*' OR immunotherapy OR 'PD-1' OR 'PD-L1') AND ('locally advanced' OR resectable OR unresectable OR neoadjuvant OR adjuvant OR perioperative OR chemoradiotherapy) AND (randomized OR randomised OR 'phase II' OR 'phase III')

Cochrane 1C:

Search concept	Search terms
Population	"head and neck squamous cell carcinoma" OR HNSCC OR "head and neck cancer"
Intervention	pembrolizumab OR nivolumab OR durvalumab OR avelumab OR atezolizumab OR "immune checkpoint inhibitor*" OR immunotherapy OR "PD-1" OR "PD-L1"

Disease setting	locally advanced OR resectable OR unresectable OR neoadjuvant OR adjuvant OR perioperative OR chemoradiotherapy
Study design	randomized OR randomised OR "phase II" OR "phase III"
Final search strategy	("head and neck squamous cell carcinoma" OR HNSCC OR "head and neck cancer") AND (pembrolizumab OR nivolumab OR durvalumab OR avelumab OR atezolizumab OR "immune checkpoint inhibitor*" OR immunotherapy OR "PD-1" OR "PD-L1") AND (locally advanced OR resectable OR unresectable OR neoadjuvant OR adjuvant OR perioperative OR chemoradiotherapy) AND (randomized OR randomised OR "phase II" OR "phase III")

Supplementary Figure S1 Funnel Plot of Comparison.

Funnel plot assessing potential publication bias among included studies for the pooled efficacy outcome.

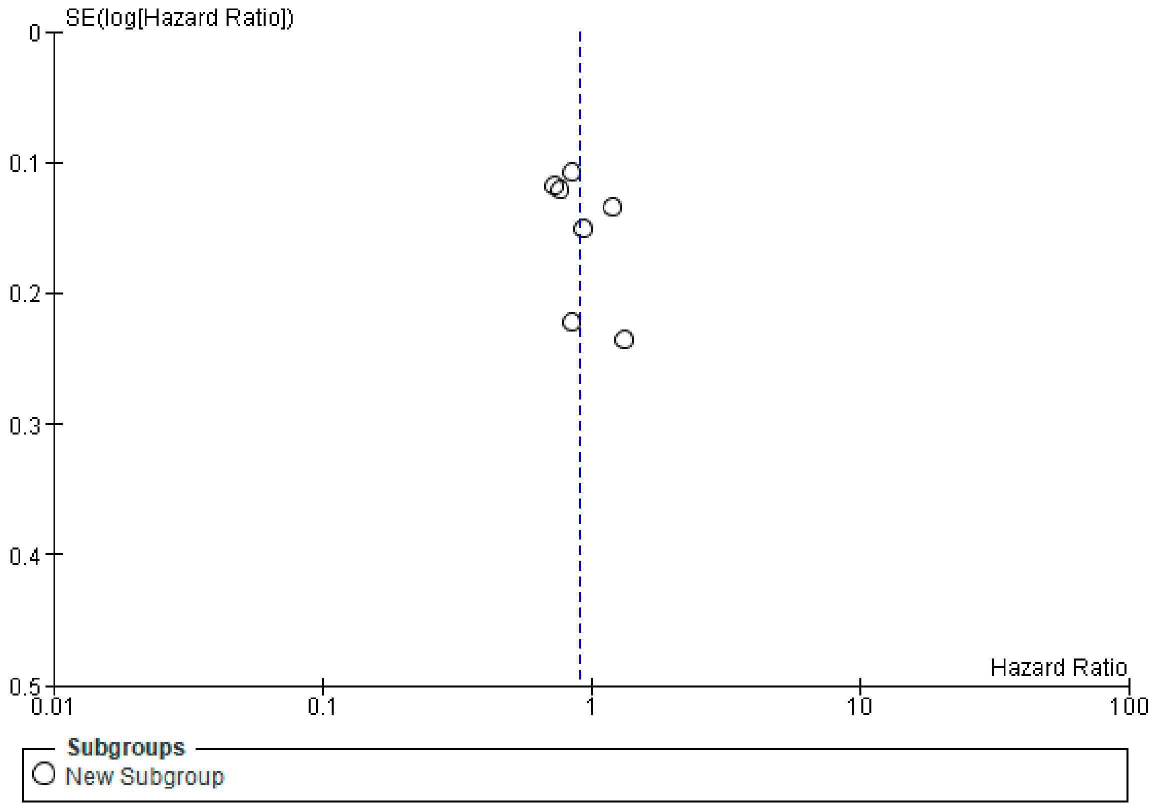

Supplementary Figure S2 Risk Of Bias Graph.
Risk of bias graph summarizing the overall percentage of studies judged as low, unclear, or high risk of bias across each Cochrane risk-of-bias domain.

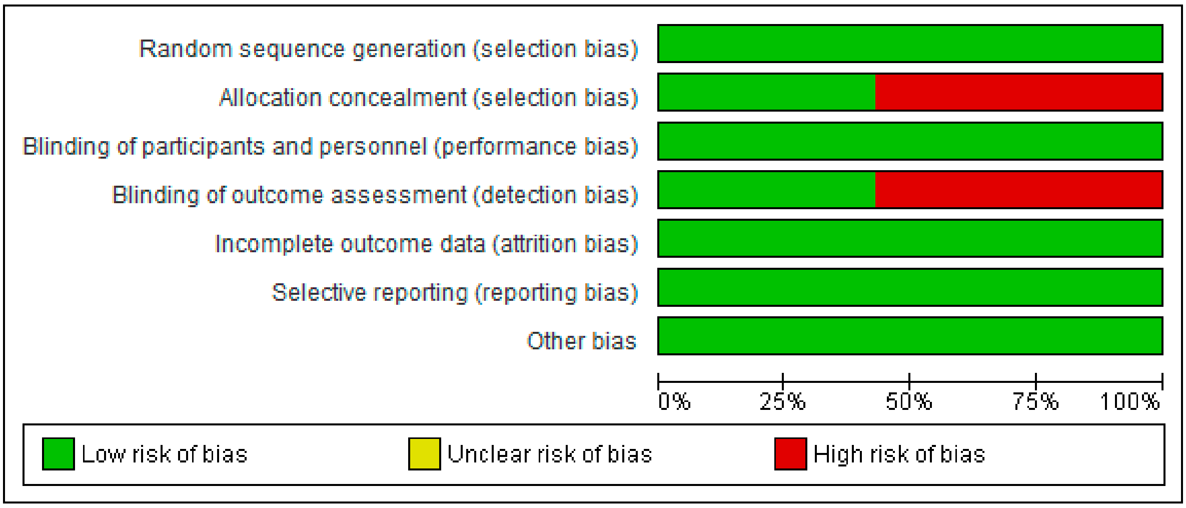

Supplementary Figure S3 Risk of Bias Summary.
Risk of bias summary showing domain-level judgments for each included randomized controlled trial.

	Random sequence generation (selection bias)	Allocation concealment (selection bias)	Blinding of participants and personnel (performance bias)	Blinding of outcome assessment (detection bias)	Incomplete outcome data (attrition bias)	Selective reporting (reporting bias)	Other bias
Bourhis 2025 NIVOPOSTOP GORTEC 2018-01	+	+	+	+	+	+	+
Haddad 2025 IMvoke010	+	+	+	+	+	+	+
Lee 2021 JAVELIN Head and Neck 100	+	−	+	−	+	+	+
Machiels 2024 KEYNOTE-412	+	−	+	−	+	+	+
Mell 2024 NRG-HN004	+	−	+	−	+	+	+
Tao 2023 GORTEC 2015-01 PembroRad	+	−	+	−	+	+	+
Uppaluri 2025 KEYNOTE-689	+	+	+	+	+	+	+

Supplementary Table S2: Summary of PD-L1 subgroup definitions and the number of PD-L1–positive and PD-L1–negative patients reported across included trials.

Trial	PD-L1 Definition	PD-L1 Positive Patients	PD-L1 Negative Patients
KEYNOTE-412	CPS ≥1	685	82
NIVOPOSTOP	CPS ≥1	544	78
IMvoke010	TAP ≥5%	315	78
NRG-HN004	CPS ≥1	127	33
PembroRad	CPS ≥1	114	11
KEYNOTE-689	CPS ≥1	682	27
JAVELIN HN100	TPS ≥25%	123	486

Supplementary Table S3: Key characteristics of included randomized controlled trials, including study phase, population, sample size, intervention, control arm, treatment setting, primary endpoint, and PD-L1 positivity definition.

Study	Phase	Population	N (Total)	Intervention	Control	Setting	Primary Endpoint	PD-L1 Positive Definition	OS Median Follow Up (Months)
KEYNOTE-412	III	LA-HNSCC, cisplatin-eligible	804	Pembrolizumab + CRT	CRT + placebo	Definitive	EFS	CPS ≥1	47.7
JAVELIN HN100	III	LA-HNSCC, cisplatin-eligible	697	Avelumab + CRT	CRT + placebo	Definitive	PFS	TPS ≥25%	16.7 in treatment arm; 16.8 in control arm
NRG-HN004	II	Cisplatin-ineligible	190	Durvalumab + RT	Cetuximab + RT	Definitive	PFS	CPS ≥1	27.6
GORTEC 2015-01 (PembroRad)	II	Cisplatin-ineligible	133	Pembrolizumab + RT	Cetuximab + RT	Definitive	LRC (PFS Secondary endpoint)	CPS ≥1	25.6 in treatment arm; 25.8 months in control arm
KEYNOTE-689	III	Resectable LA-HNSCC	363	Perioperative pembrolizumab	Surgery+adjuvant RT +/- cisplatin	Perioperative	EFS	CPS ≥1	38.3
NIVOPOSTOP (GORTEC 2018-01)	III	High-risk resected LA-HNSCC	680	Nivolumab + adjuvant RT/CRT	RT/CRT	Postoperative	DFS	CPS ≥1	30.3
IMvoke010	III	Post-definitive LA-HNSCC	406	Atezolizumab maintenance	Placebo	Maintenance	EFS	TAP ≥5%	46.5
